# Micheliolide Alleviates Hepatic Fibrosis by Inhibiting Autophagy in Hepatic Stellate Cells via the TrxR1/2-Mediated ROS/MEK/ERK Pathway

**DOI:** 10.3390/ph18030287

**Published:** 2025-02-20

**Authors:** Yi Liu, Ling Yao, Yuanyuan Liu, Yunheng Yang, Ailing Liang, Honglin He, Yao Lei, Wenfu Cao, Zhiwei Chen

**Affiliations:** 1 Key Laboratory of Traditional Chinese Medicine for Prevention and Cure of Metabolic Diseases, College of Traditional Chinese Medicine, Chongqing Medical University, Chongqing 400016, China; 2 College of Traditional Chinese Medicine, Chongqing University of Chinese Medicine, Chongqing 402760, China; 3 Department of Radiological Medicine, School of Basic Medical Sciences, Chongqing Medical University, Chongqing 400016, China

**Keywords:** micheliolide, hepatic fibrosis, reactive oxygen species, MEK/ERK pathway, TrxR

## Abstract

**Background**: Hepatic fibrosis is a major global health issue without an optimal drug treatment, highlighting the urgent need to find effective therapies. This study aimed to clarify the role and mechanism of micheliolide in treating hepatic fibrosis. **Methods**: The efficacy of MCL was evaluated in a mouse model of CCl_4_-induced hepatic fibrosis. LX-2 cells were subjected to MCL treatment, and subsequent changes in fibrosis markers, autophagy, and the MEK/ERK pathway were analyzed using transcriptomics and Western blotting. The interaction between MCL and TrxR1 or TrxR2 were validated using cellular thermal shift assays (CETSA) and drug affinity responsive target stability (DARTS) assays. **Results**: Our findings indicated that MCL significantly alleviated CCl_4_-induced hepatic fibrosis, improved liver function, and downregulated the expression of fibrosis markers. Additionally, MCL significantly inhibited LX-2 cell activation by suppressing cell proliferation, extracellular matrix (ECM) production, and autophagy, while activating the MEK/ERK pathway. Moreover, MCL elevated intracellular and mitochondrial reactive oxygen species (ROS) levels, reduced mitochondrial membrane potential, and altered mitochondrial morphology. The ROS scavenger N-acetylcysteine (NAC) attenuated MCL-induced MEK/ERK pathway activation and increased collagen type I alpha 1 (COL1A1) and fibronectin (FN) expression. Further analysis confirmed that MCL directly interacts with TrxR1 and TrxR2, leading to the inhibition of their enzymatic activities and the induction of ROS generation. Ultimately, MCL attenuated the fibrotic process and autophagic flux in LX-2 cells. **Conclusions**: The findings of our study confirmed that MCL has the potential to alleviate hepatic fibrosis, thereby introducing a novel candidate drug and therapeutic strategy for management of this condition.

## 1. Introduction

Around two million people die each year due to chronic liver disease, with cirrhosis accounting for nearly half of all deaths [1]. Cirrhosis stems from hepatic fibrosis, so preventing fibrosis is crucial. Hepatic fibrosis, marked by excessive ECM accumulation, arises from chronic liver injuries such as viral infections, alcoholic liver disease, and non-alcoholic causes [2]. The activation of hepatic stellate cells (HSCs) is the main catalyst of fibrosis [3]. Chronic liver injury converts quiescent, vitamin A-rich HSCs into proliferative myofibroblast-like cells, resulting in excessive ECM production, such as collagen, and contributing to hepatic fibrosis [4]. The inhibition of HSC activation has become a key therapeutic approach in the treatment of hepatic fibrosis.

Autophagy is a cellular process that eliminates excess proteins and organelles by encapsulating them in autophagosomes, which then merge with lysosomes for degradation, maintaining cellular homeostasis and responding to metabolic stress [5]. Autophagy exerts a beneficial influence on HSC activation. Evidence demonstrates that autophagic flux is heightened in activated HSCs [6], facilitating their transformation into a myofibroblastic phenotype. Autophagy facilitates HSC activation by degrading lipid droplets in quiescent HSCs [7]. Beyond lipid peroxidation, the autophagy substrate p62 has been identified as a regulatory mechanism in HSC activation [8]. Consequently, regulating autophagy has become an important alleviating strategy for addressing hepatic fibrosis.

ROS are generated during biochemical processes in organelles such as the endoplasmic reticulum and mitochondria [9]. Elevated ROS levels can induce cellular apoptosis, leading to the death of HSCs [10]. In mammals, the thioredoxin system, comprising thioredoxin, thioredoxin reductase (TrxR), and reduced NADPH, function as an essential antioxidant defense mechanism. Research has demonstrated a close relationship between the hyperactivity of the thioredoxin system and the pathogenesis of various cancers [11]. Currently, several TrxR inhibitors with anticancer properties have been identified [12,13,14]. These inhibitors enhance ROS accumulation, disrupt cellular redox balance, and trigger apoptosis in cancer cells. A noteworthy inhibitor is butaselen, which shows promise in treating pulmonary fibrosis [15]. Consequently, the evaluation of TrxR inhibitors for their potential therapeutic applications in hepatic fibrosis warrants significant attention.

Micheliolide is a guaiane-type sesquiterpene lactone derived from *Michelia compressa* and *Michelia champaca* and can also be synthesized from parthenolide [16]. MCL demonstrates multiple biological effects, such as anti-inflammatory [17], anticancer [18], and neuroprotective effects [19]. MCL demonstrates antitumor activity in hepatocellular carcinoma by triggering ROS-induced endoplasmic reticulum stress and facilitating immunogenic cell death [20]. The derivative DMAMCL has been demonstrated to significantly mitigate peritoneal fibrosis and inhibit ECM deposition in a peritoneal dialysis mouse model by regulating autophagy [21]. One recent study indicates that MCL mitigates renal fibrosis by disrupting the IL-11 and IL-11Rα1 interaction [22]. Despite the increasing interest in the anti-fibrotic potential of MCL, there is currently no research exploring its efficiency in the context of hepatic fibrosis.

The present research aimed to evaluate the efficiency of MCL in mitigating hepatic fibrosis and to clarify the underlying molecular mechanisms. The research specifically investigated the mechanism through which MCL enhances reactive oxygen species levels, impeding autophagic flux by directly interacting with TrxR1 and TrxR2.

## 2. Results

### 2.1. Micheliolide Alleviated Hepatic Fibrosis in CCl_4_-Treated Mouse Mode

**Figure 1:**
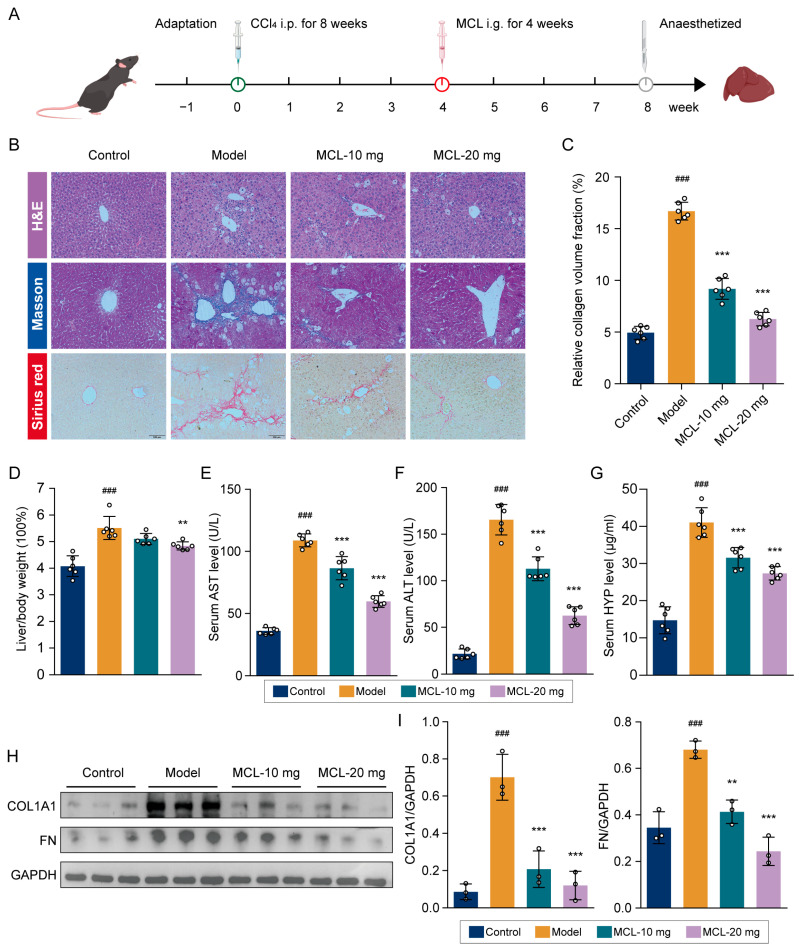
Effects of MCL on CCl_4_-induced hepatic fibrosis in vivo. (**A**) Schematic of the experimental procedure for animal treatment. (**B**) Representative photographs of liver sections were stained with H&E, Masson, and Sirius Red. (**C**) Statistical analysis of collagen volume fraction based on Masson staining. (**D**) The liver-to-body weight ratio analysis. (**E**–**G**) The serum levels of AST, ALT, and HYP in mice. (**H**) Western blot analysis of COL1A1 and FN in liver tissue. (**I**) Histogram showing quantification of bands in (**H**). ^###^ *p* < 0.001 vs. control group. ** *p* < 0.01 and *** *p* < 0.001 vs. model group.

This research employed a carbon tetrachloride (CCl_4_)-treated model to examine the effects of MCL on hepatic fibrosis. The experimental workflow is depicted in Figure 1A. Liver tissue alterations were evaluated using hematoxylin and eosin (H&E), Masson, and Sirius Red staining techniques (Figure 1B). The H&E results indicated that the normal liver lobular architecture was preserved. The livers showed vacuolar changes and disrupted lobular structures in the model group. However, treatment with MCL led to a marked decrease in inflammatory cell infiltration and hepatocyte degeneration, consequently ameliorating hepatic fibrosis. Histological analyses using Masson and Sirius Red staining revealed an increase in and expansion of collagen fibers extending towards the portal regions in the model group. In contrast, the MCL treatment group indicated notable improvements in hepatocyte morphology and reduced collagen fiber deposition. Masson staining statistical analysis demonstrated a significant reduction in the collagen volume fraction due to MCL (Figure 1C).

The liver-to-body weight ratio is a crucial indicator of murine liver health. The model group exhibited a significantly higher liver-to-body weight ratio than the control group, which decreased dose-dependently with MCL treatment (Figure 1D). Serum markers of liver function, such as aspartate transaminase (AST), alanine transaminase (ALT), and hydroxyproline (HYP), were notably reduced in the MCL treatment group (Figure 1E–G), indicating enhanced liver function. COL1A1 and FN are recognized as key biomarkers of fibrosis. We investigated the impact of MCL on COL1A1 and FN expression levels. Our findings indicated that MCL markedly suppressed COL1A1 and FN expression in liver tissue (Figure 1H,I), suggesting its considerable therapeutic potential for hepatic fibrosis treatment.

### 2.2. Micheliolide Inhibited the Proliferation of HSCs

**Figure 2:**
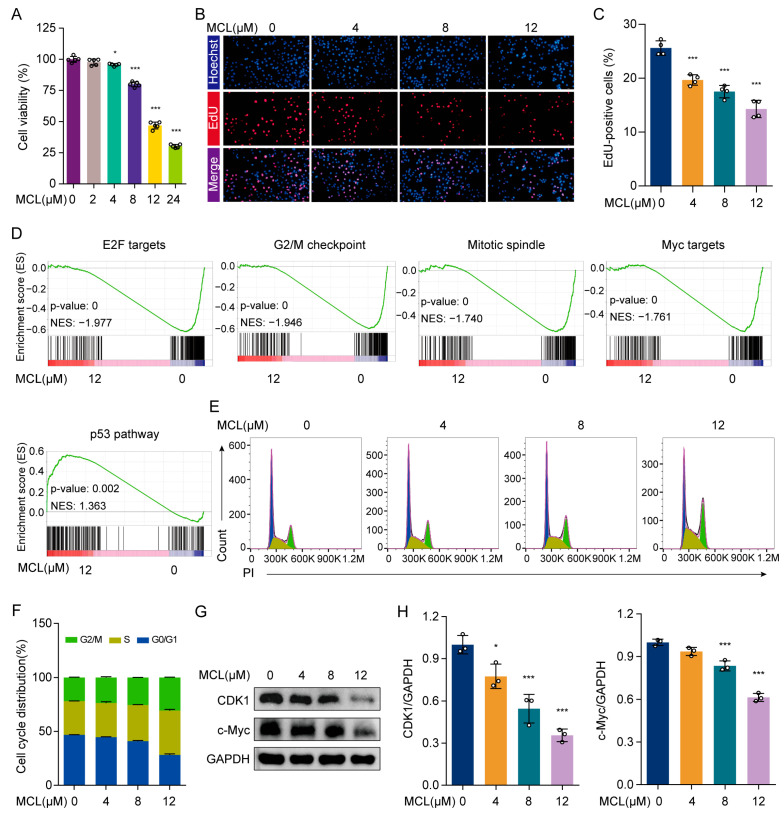
Effects of MCL on LX-2 cell proliferation in vitro. (**A**) Relative cell viability of LX-2 cells treated with MCL. (**B**) Analysis of cell proliferation using EdU labeling. (**C**) The percentage analysis of EdU-positive LX-2 cells. (**D**) GSEA enrichment plots of proliferation-related gene sets. (**E**) Analysis of cell cycle using flow cytometry. (**F**) Graph represents quantitative data of cell cycle distribution. (**G**) Western blot analysis of CDK1 and c-Myc in LX-2 cells. (**H**) Histogram showing quantification of bands in (**G**). * *p* < 0.05 and *** *p* < 0.001 vs. 0 μM.

To assess the impact of MCL on HSC proliferation, we performed CCK-8 assays on LX-2 cells. The result indicated a notable reduction in the viability of LX-2 cells as the concentration of MCL increased (Figure 2A). The half-maximal inhibitory concentration (IC50) of MCL was 13.69 μM at 24 h in LX-2 cells. EdU (5-ethynyl-2′-deoxyuridine), a thymidine analog, was used to integrate into the DNA of proliferating cells during the synthesis phase. As illustrated in Figure 2B,C, the proportion of EdU-positive cells was significantly diminished following MCL treatment, thereby corroborating the inhibitory effect of MCL on HSC proliferation. Furthermore, GSEA indicated a significant downregulation of four gene sets closely linked to cell proliferation following MCL treatment. The gene sets comprise E2F targets, the G2M checkpoint, Myc targets, and the mitotic spindle. Concurrently, the gene set associated with the p53 pathway, which is critical in proliferation regulation, was markedly upregulated (Figure 2D).

Flow cytometry analysis revealed MCL-induced cell cycle arrest at the G2/M phase, providing insight into its molecular mechanism of inhibiting cell proliferation (Figure 2E,F). Subsequent investigations focused on key regulators of the G2/M checkpoint, specifically CDK1, as well as a major regulator of Myc targets, c-Myc, to assess the impact of MCL on cell cycle progression. The data demonstrated that MCL notably decreased the protein levels of CDK1 and c-Myc (Figure 2G,H). In summary, MCL effectively inhibits the proliferation of HSCs.

### 2.3. Micheliolide Repressed the Activation of HSCs

**Figure 3:**
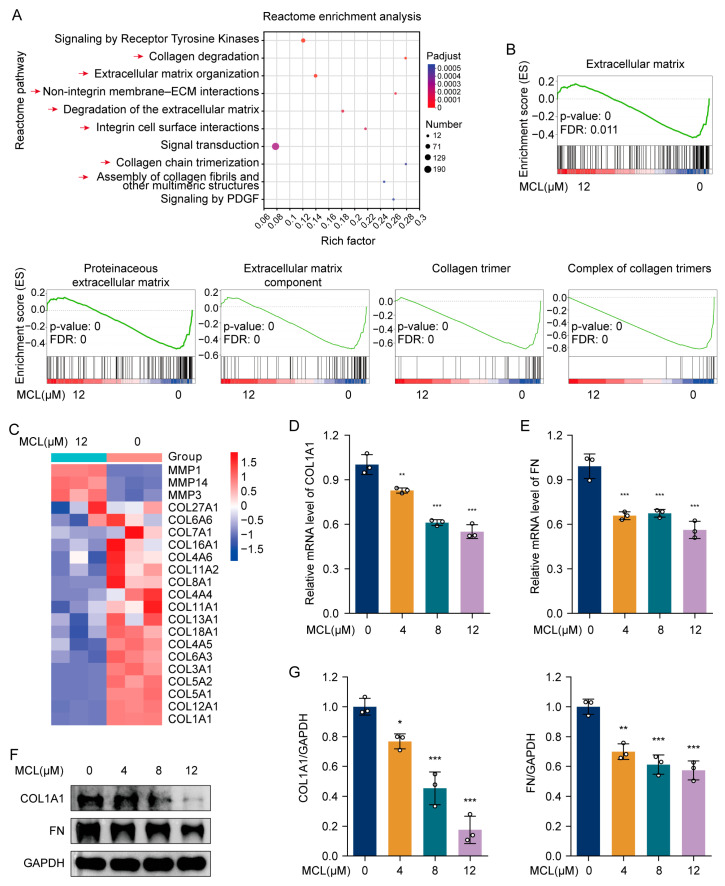
Effects of MCL on LX-2 cell activation in vitro. (**A**) Reactome enrichment analysis of differentially expressed genes. Red arrows: ECM-related processes. (**B**) The gene sets are associated with ECM. (**C**) The heatmap shows the relative levels of MMPs and collagen-encoding genes. (**D**,**E**) Relative mRNA levels of COL1A1 and FN in LX-2 cells. (**F**) Western blot analysis of COL1A1 and FN in LX-2 cells. (**G**) Histogram showing quantification of bands in (**F**). * *p* < 0.05, ** *p* < 0.01 and *** *p* < 0.001 vs. 0 μM.

HSC activation occurs during hepatic fibrosis, resulting in elevated ECM production and accumulation. The LX-2 cell line, originating from human HSCs [23], preserves essential features of activated HSCs and was utilized to evaluate the impact of MCL on HSC activation. Transcriptome sequencing and Reactome enrichment analysis highlighted several key ECM-related processes (Figure 3A). The processes included collagen degradation, ECM organization, non-integrin membrane–ECM interactions, degradation of the ECM, integrin cell surface interactions, collagen chain trimerization, and the assembly of collagen fibrils and other multimeric structures. Additionally, gene sets associated with ECM were downregulated, particularly those involved in collagen trimer formation and its complexes (Figure 3B). Heatmap analysis revealed that following MCL treatment, mRNA levels of MMP1, MMP14, and MMP3 were upregulated, indicating an increase in collagen degradation, whereas the expression of several collagen-encoding genes was downregulated (Figure 3C). qPCR analysis revealed a notable decrease in COL1A1 and FN mRNA levels after MCL treatment (Figure 3D,E). This observation was corroborated by Western blot analysis (Figure 3F,G). Collectively, these findings suggest that MCL inhibits HSC activation by downregulating the expression of ECM components and facilitating collagen degradation.

### 2.4. Micheliolide Impaired Autophagic Flux of HSCs

**Figure 4:**
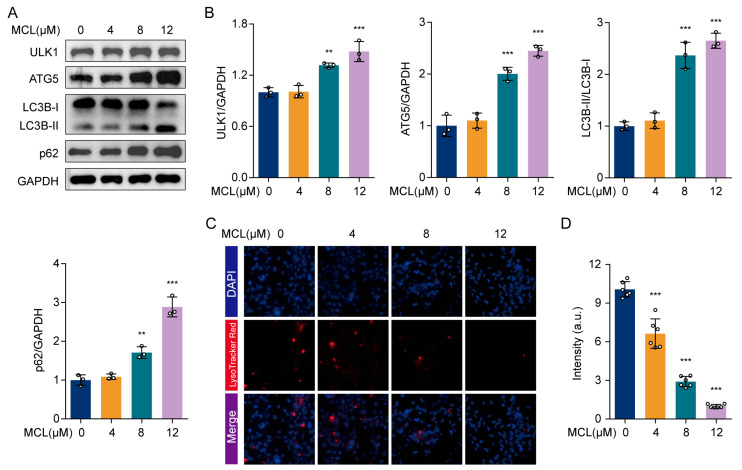
Effects of MCL on LX-2 cell autophagy. (**A**) Western blot analysis of ATG5, LC3B, and p62 in LX-2 cells. (**B**) Histogram showing quantification of bands in (**A**). (**C**) Lysosomes of LX-2 cells as stained with LysoTracker Red. (**D**) Relative level of fluorescence intensity. ** *p* < 0.01 and *** *p* < 0.001 vs. 0 μM.

During hepatic fibrosis resolution, activated HSCs can undergo programmed cell death or revert to a quiescent state [4]. Our assay indicated that MCL can trigger apoptosis in LX-2 cells, although the minimal number of apoptotic cells implies that apoptosis is not the main mechanism through which MCL inhibits HSC activation. Studies have demonstrated that autophagy supplies energy for the activation of HSCs, and inhibiting autophagy can retard the progression of hepatic fibrosis [6,24]. To clarify MCL’s impact on autophagy, we investigated the levels of proteins associated with autophagy in LX-2 cells. The data demonstrated that MCL dose-dependently increased the levels of the autophagy-promoting proteins ULK1 and ATG5 (Figure 4A,B). Additionally, MCL promoted the conversion of LC3B-I to LC3B-II. Autophagy is a complex, multi-step physiological process, and an increase in LC3B-II is generally interpreted as indicative of autophagosome formation rather than the completion of autophagy [25]. Notably, MCL significantly increased p62 levels (Figure 4A,B). Under normal conditions, p62 is degraded in the autolysosome. Its accumulation indicates inhibited autophagy, particularly a disruption in the late autophagic flux involving lysosomal function [26,27]. Lysosomes require an acidic environment for the activity of acidic hydrolases and hydrolytic processes. Lyso-Tracker Red serves as an acidic pH marker for lysosomes. Lyso-Tracker Red staining demonstrated that increasing MCL concentration progressively reduced the red signal (Figure 4C,D), indicating decreased lysosomal acidity and impaired lysosomal function in LX-2 cells, ultimately halting late-stage autophagy.

### 2.5. Micheliolide Activated the MEK/ERK Pathway

**Figure 5:**
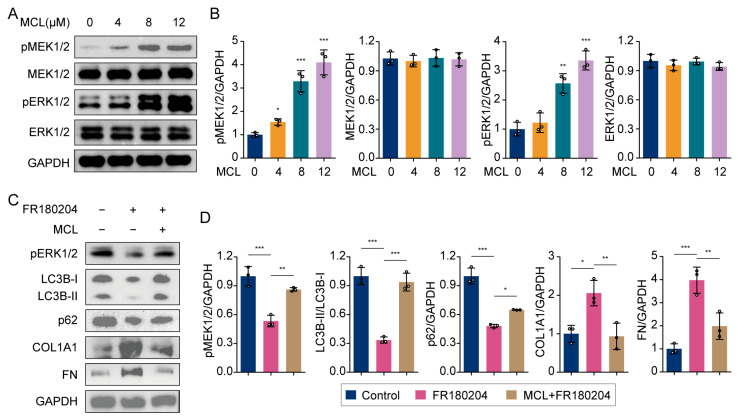
Effects of MCL on the MEK/ERK pathway. (**A**) Western blot analysis of pMEK1/2, MEK1/2, pERK1/2, and ERK1/2 in LX-2 cells. (**B**) Histogram showing quantification of bands in (**A**). * *p* < 0.05, ** *p* < 0.01 and *** *p* < 0.001 vs. 0 μM. (**C**) Western blot analysis of pERK1/2, LC3B, p62, COL1A1, and FN in LX-2 cells. (**D**) Histogram showing quantification of bands in (**C**). * *p* < 0.05, ** *p* < 0.01 and *** *p* < 0.001.

Prior studies indicate that activating the MEK/ERK pathway leads to elevated p62 levels and a higher LC3B-II/LC3B-I ratio [28]. We proposed that MCL could influence autophagy in HSCs by activating the MEK/ERK pathway. Subsequent analyses indicated that the total protein levels of MEK1/2 and ERK1/2 remained constant following MCL treatment, whereas their phosphorylation levels significantly increased (Figure 5A,B). This observation suggests that MCL activates the MEK/ERK pathway. We subsequently analyzed the impact of MCL on autophagy and HSC activation under conditions of MEK/ERK pathway inhibition. Our findings revealed that ERK1/2 inhibition led to a reduction in LC3B-II and p62 levels, concomitant with an increase in COL1A1 and FN expression (Figure 5C,D). Further analysis demonstrated that co-treatment with MCL and an ERK1/2 inhibitor (FR180204) partially mitigated these protein changes (Figure 5C,D). These results suggest that MCL modulates autophagy and inhibits HSC activation by activating the MEK/ERK pathway.

### 2.6. Micheliolide Induced ROS Generation and Activated the MEK/ERK Pathway

**Figure 6:**
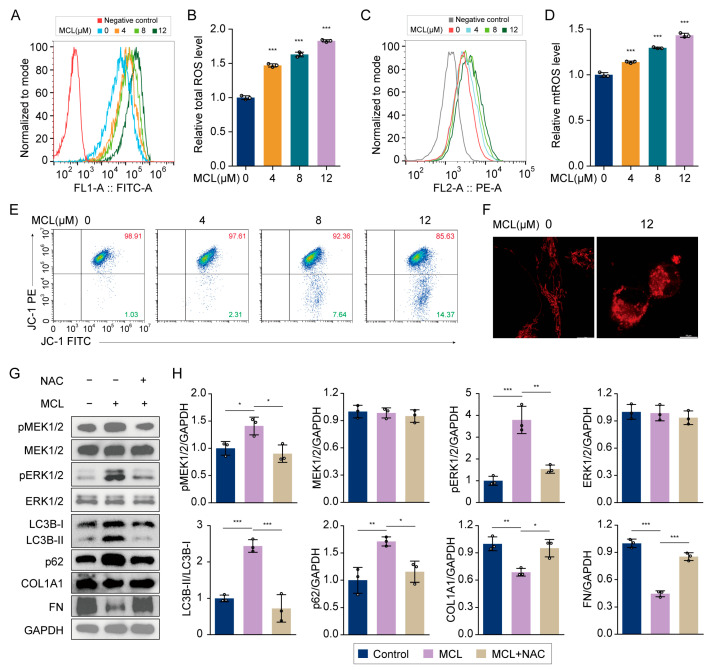
Effects of MCL on ROS and mitochondria. (**A**) Flow cytometric measurement of ROS levels in LX-2 cells. (**B**) Relative total ROS levels in (**A**). (**C**) Flow cytometric measurement of ROS levels in mitochondria. (**D**) Relative mtROS levels in (**C**). (**E**) Flow cytometric analysis of mitochondrial membrane potential in LX-2 cells. (**F**) Morphological observation of mitochondria using confocal microscopy. (**G**) Western blot analysis of pMEK1/2, MEK1/2, pERK1/2, ERK1/2, LC3B, p62, COL1A1, and FN in LX-2 cells. (**H**) Histogram showing quantification of bands in (**G**). * *p* < 0.05, ** *p* < 0.01 and *** *p* < 0.001.

Previous studies have demonstrated that ROS activate the MEK/ERK pathway [28]. In light of this, we evaluated ROS levels in LX-2 cells following treatment with MCL. Our findings revealed that MCL significantly elevated the total ROS levels in LX-2 cells in a dose-dependent manner (Figure 6A,B). Given that mitochondria are the primary source of ROS within cells, we further investigated the effect of MCL on mitochondrial ROS levels and observed a marked increase (Figure 6C,D). Flow cytometry analysis showed a reduction in mitochondrial membrane potential induced by MCL (Figure 6E). Moreover, confocal microscopy indicated that MCL treatment resulted in morphological abnormalities in LX-2 cell mitochondria (Figure 6F), suggesting mitochondrial dysfunction.

We examined the effects of the antioxidant NAC on LX-2 cells subjected to MCL exposure. Our findings indicated that NAC treatment did not alter the total protein levels of MEK and ERK; however, it mitigated the MCL-induced phosphorylation of these proteins (Figure 6G,H). Additionally, NAC attenuated the increase in LC3B-II and p62 levels and counteracted the reduction in COL1A1 and FN levels. In conclusion, MCL activates the MEK/ERK pathway through the elevation of ROS, and NAC can partially ameliorate the inhibitory effects of MCL on HSC activation, underscoring the pivotal role of ROS in the mechanism of MCL action.

### 2.7. Micheliolide Directly Interacted with TrxR1 and TrxR2 in HSCs

**Figure 7:**
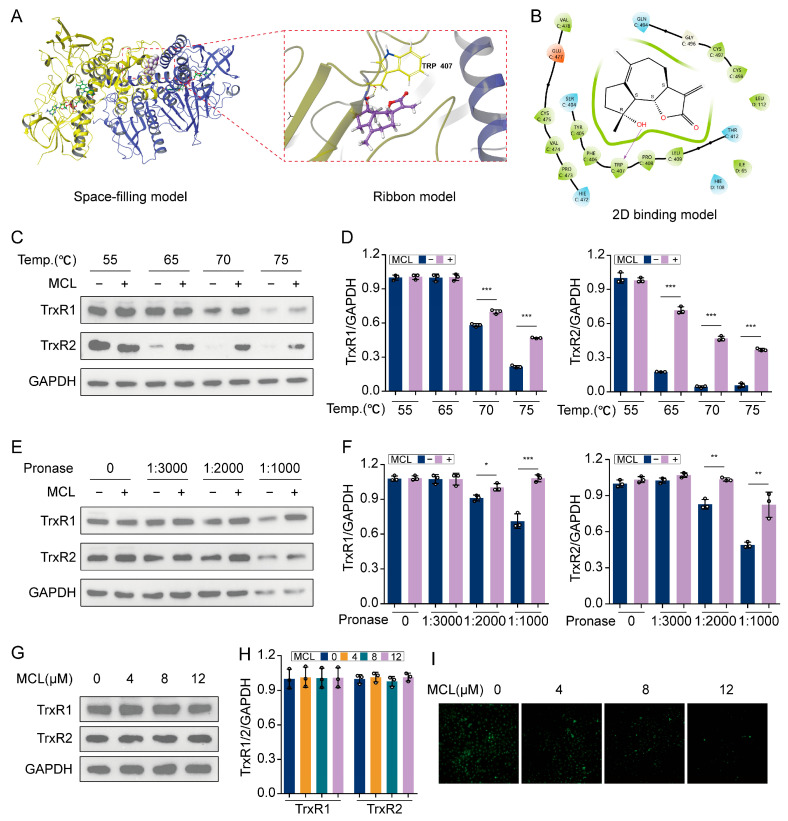
MCL targets TrxR1 and TrxR2 in LX-2 cells. (**A**) The space-filling and ribbon models of MCL and human TrxR1 complexes. (**B**) The 2D binding model of MCL and human TrxR1 complexes. (**C**) Western blot analysis of TrxR1 and TrxR2 in CETSA. (**D**) Histogram showing quantification of bands in (**C**). (**E**) Western blot analysis of TrxR1 and TrxR2 in DARTS. (**F**) Histogram showing quantification of bands in (**E**). (**G**) Western blot analysis of TrxR1 and TrxR2 in LX-2 cells. (**H**) Histogram showing quantification of bands in (**G**). (**I**) The activity of TrxR was detected by a TRFS green probe in LX-2 cells. * *p* < 0.05, ** *p* < 0.01 and *** *p* < 0.001.

Thioredoxin reductase (TrxR) is a crucial enzyme that reduces thioredoxin, aiding in the elimination of excess ROS [29]. Mammals have three TrxR subtypes: TrxR1, TrxR2, and TrxR3. TrxR1 is mainly found in the cytoplasm, TrxR2 in mitochondria, and TrxR3 is confined to the testes. Previous studies have demonstrated that MCL interacts with TrxR1 in hepatocellular carcinoma and HeLa cells, increasing ROS production [20,30]. However, the interaction between MCL and TrxR1 or TrxR2 in HSCs is not well understood. Initially, molecular docking revealed that MCL binds deeply within the active pocket of the human TrxR1 protein (PDB ID: 2J3N, Chains C and D) (Figure 7A). The Extra Precision (XP) docking score was −4.441, and the MM-GBSA analysis showed a binding free energy of −30.82 kcal/mol, indicating a stable interaction. Further analysis revealed that the binding pocket of MCL includes key residues CYS497 and CYS498, crucial for TrxR activity, and forms a hydrogen bond with TRP407 (Figure 7B).

To confirm the interaction between MCL and TrxR, we used CETSA, which detects drug-target binding in live cells by measuring increased thermal stability. The results showed that MCL provided significant protection to TrxR1 at 70 and 75 °C, whereas enhanced protection for TrxR2 was observed starting at 65 °C (Figure 7C,D). These findings suggest that MCL effectively binds to both targets in HSCs. We performed a DARTS assay, which operates on the principle that small molecule drug binding reduces target proteolysis. The results demonstrated that at a 1:1000 and 1:2000 protease ratio, MCL reduced proteolysis of TrxR1 and TrxR2 (Figure 7E,F), confirming its high affinity for both targets. We examined whether MCL influences the expression of TrxR1 and TrxR2. The results showed that MCL treatment did not alter TrxR1 or TrxR2 protein levels (Figure 7G,H). However, using a probe to measure TrxR activity, we observed a significant decrease in activity with higher MCL dosage (Figure 7I). This observation suggests that MCL acts as an inhibitor of TrxR activity, consequently leading to elevated ROS production.

## 3. Discussion

**Figure 8:**
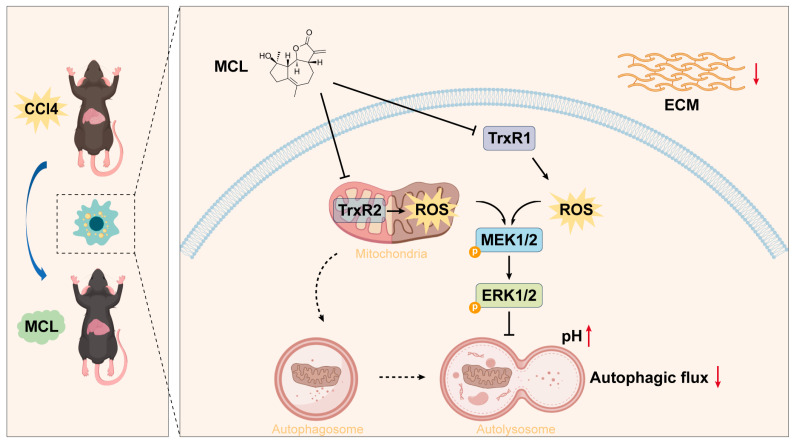
Schematic representation of the molecular mechanism by which MCL alleviates hepatic fibrosis by inhibiting autophagy in hepatic stellate cells via the TrxR1/2-mediated ROS/MEK/ERK pathway.

MCL, a sesquiterpene lactone featuring an α-methylene-γ-lactone structure, can be synthesized from parthenolide [16]. Parthenolide is identified as a crucial precursor in the development of antifibrotic agents [31]. Notably, MCL demonstrates enhanced stability and solubility in comparison to parthenolide [32,33]. Research has primarily focused on the anticancer effects of MCL in different cancers [34], including hepatocellular carcinoma [35], breast cancer [36], and pancreatic cancer [18]. However, research exploring the association of MCL with chronic diseases remains limited, with no reported studies specifically addressing its connection to hepatic fibrosis. This study suggested that MCL effectively ameliorated hepatic fibrosis by targeting TrxR1 and TrxR2, thereby enhancing ROS production, activating the MEK/ERK pathway, and inhibiting HSC autophagy (Figure 8). These findings highlight the significant prospect of MCL in antifibrotic therapy.

The liver is a highly dynamic metabolic organ that plays a crucial role in energy metabolism and protein synthesis. Autophagy is essential for maintaining liver tissue homeostasis by degrading damaged organelles and misfolded proteins [37]. Dysregulation of autophagy can result in various chronic liver diseases [5]. Research suggests that active autophagy facilitates the trans-differentiation of quiescent HSCs into myofibroblast-like cells [38]. Therefore, the inhibition of autophagy in HSCs can effectively suppress hepatic fibrosis. Our research clarified MCL’s distinct dual function in autophagy regulation. Specifically, MCL enhanced the expression of ATG5, a critical component in autophagosome formation that interacts with ATG12 and ATG16L1 to collectively facilitate autophagosome assembly [39]. Elevated levels of ATG5 indicated that MCL promotes the early stages of autophagy, a notion further supported by the observed increase in the LC3B-II/LC3B-I ratio. The initiation of early autophagy is likely due to an increase in damaged mitochondria. Concurrently, MCL also upregulated the expression of p62, an adaptor protein that facilitates interactions between autophagosomes and their substrates and is subsequently degraded within autolysosomes during the later stages of autophagy [40]. The accumulation of p62 suggests an impairment in autophagic flux. Lysosomes, characterized by their acidic milieu, demonstrate diminished acidity upon exposure to MCL, suggesting a decline in lysosomal hydrolase activity. This reduction in hydrolase function is posited to be the principal factor underlying the accumulation of p62 or the interruption of autophagic flux. These results offer new insights that could inform the development of innovative antifibrotic strategies.

ROS are acknowledged as pivotal mediators in the facilitation of migration, proliferation, and overexpression of ECM components in HSCs [41,42]. Previous study has demonstrated that ROS positively influence autophagy in HSCs [43]. Recent studies indicate that ROS hinder autophagic flux by causing lysosomal membrane permeabilization (LMP) and disrupting lysosomal degradation processes [44]. These findings provide a deeper understanding of the complex mechanisms by which ROS regulate autophagy. MCL, a TrxR1 inhibitor, suppresses hepatocellular carcinoma cell proliferation by increasing ROS levels [20]. In the present study, we observed impaired mitochondrial function and increased ROS production, indicating that MCL likely inhibits targets beyond cytoplasmic TrxR1. Interestingly, our findings demonstrated that MCL also targets TrxR2, thereby contributing to the observed elevation in mitochondrial ROS. TrxR1 and TrxR2 share an 84% similarity in their amino acid sequences [45], resulting in analogous spatial structures and catalytic mechanisms. This similarity underpins MCL’s capacity to concurrently target both TrxR1 and TrxR2. Historically, therapeutic strategies targeting TrxR have predominantly focused on oncological diseases [46], with relatively fewer investigations addressing fibrotic conditions. However, this trend is shifting. Currently, clinical trials are underway in China (Registration No.: CTR20222921) to assess the efficacy of combining standard therapy with the TrxR inhibitor butaselen for the treatment of fibrotic interstitial lung disease. This progress underscores TrxR’s potential as a therapeutic target for diseases associated with fibrosis. Our research underscores the potential of MCL-targeted TrxR therapy in the treatment of hepatic fibrosis; however, its clinical application is constrained by its limited water solubility and short half-life. The utilization of nanoparticles can enhance drug stability and targeting capabilities, facilitating selective delivery to affected tissues and minimizing systemic side effects [47]. Empirical evidence suggests that nanoparticle-mediated delivery of MCL improves serum stability and effectively reduces leukemic stem cell burden in vivo [48]. Consequently, the development of targeted nanoparticles encapsulating MCL for the treatment of hepatic fibrosis presents a promising therapeutic avenue.

## 4. Materials and Methods

### 4.1. Reagents

Micheliolide (purity > 99%) was procured from MedChemExpress (Princeton, NJ, USA). Dimethyl sulfoxide was sourced from Beyotime (Shanghai, China). Fetal bovine serum (FBS) and DMEM medium were obtained from Vivacell (Shanghai, China). The BCA protein assay kit was obtained from Beyotime (Shanghai, China). Trizol and SYBR reagents were obtained from Takara (Kyoto, Japan).

### 4.2. Experimental Animals

Forty male C57BL/6J mice were sourced from Chongqing Medical University and acclimated for one week. Participants were allocated to a control group (n = 10) or a group treated with carbon tetrachloride (CCl_4_) (n = 30). The CCl_4_ group received biweekly intraperitoneal injections of 20% CCl_4_ in corn oil for eight weeks. After four weeks, this group was further subdivided into a model group and two drug treatment groups. The experimental protocol administered MCL to two drug treatment groups, each consisting of ten mice, at dosages of 10 mg/kg/day and 20 mg/kg/day via gavage. Both the control and model groups received a 0.5% CMC-Na solution. In the ninth week, mice were euthanized under deep anesthesia, and their liver weights were recorded.

### 4.3. Liver Histopathology

Tissue samples were taken from the edge of the largest liver lobe. One-third of the samples were fixed in 4% paraformaldehyde for 48 h, followed by dehydration, paraffin embedding, and sectioning at thickness of 4 μm. Liver sections were stained with hematoxylin and eosin (H&E), Masson, and Sirius Red. The stained sections were examined and quantitatively analyzed utilizing an optical microscope in conjunction with ImageJ software (Version: 1.45).

### 4.4. Cell Viability Assay

The LX-2 cell line was obtained from Cellcook Biotech Co., Ltd. (Guangzhou, China), and was authenticated by both Cellcook Biotech Co., Ltd. and FuHeng Cell Center (Shanghai, China). The viability of the LX-2 cells was assessed using the Cell Counting Kit-8 (CCK-8) (TargetMol, Shanghai, China). Cells were seeded in a 96-well plate and exposed to different MCL concentrations. After 24 h, 10 μL of CCK-8 was added and incubated for 3 h. Absorbance was then read at 450 nm with a microplate reader (BioTek, Winooski, VT, USA).

### 4.5. EdU Assay

The impact of MCL on LX-2 cell proliferation was evaluated using the BeyoClick™ EdU Kit (Beyotime, Shanghai, China). Various MCL concentrations were applied to LX-2 cells for 48 h. EdU was introduced for 2 h, followed by a 30-min Click reaction. Fluorescence was detected using a microscope (Olympus IX73, Tokyo, Japan).

### 4.6. Flow Cytometry Analysis of Cell Cycle

After 24 h of MCL treatment, LX-2 cells were digested with trypsin, centrifuged at 1000× *g* for 5 min, and washed with PBS. After 30 min of fixation in 70% ethanol, the cells were washed with PBS at 4 °C, propidium iodide was added, and the cells were incubated for 30 min at 37 °C. The cell cycle distribution was analyzed using a flow cytometer (Beckman Coulter Inc., Brea, CA, USA).

### 4.7. Quantitative Real-Time Polymerase Chain Reaction (qPCR)

LX-2 cells treated with MCL were harvested, followed by the addition of RNAiso Plus (TaKaRa, Dalian, China) and chloroform. After mixing thoroughly, cells were centrifuged at 12,000× *g* for RNA extraction. After transferring the supernatant, isopropanol was added, and the solution mixed. Once the pellet was centrifuged, it was washed with 75% ethanol, dried, and resuspended in DEPC-treated water. RNA concentration was measured. For PCR, initial denaturation occurred at 98 °C for 30 s, then 35 cycles of 98 °C for 5 s and 60 °C for 30 s, concluding with a final melt curve analysis. GAPDH was the mRNA expression control. The specific primer sequences utilized were as previously reported [49].

### 4.8. Western Blot Analysis

Proteins were extracted with RIPA buffer, quantified using a BCA Assay Kit (Beyotime, Shanghai, China), separated on 10% SDS-PAGE, and transferred to PVDF membrane. After blocking the membranes in nonfat milk, primary antibodies were incubated overnight at 4 °C. The secondary antibodies were incubated for two hours. Protein detection utilized a highly sensitive chemiluminescent substrate and was visualized with a ChemiDoc Imaging System (Bio-Rad, Hercules, CA, USA). GAPDH served as an internal reference for normalizing expression levels. The study employed the following antibodies: COL1A1 (ET1609-68, HUABIO, 1:1000), FN (A12932, ABclonal, 1:1000), c-Myc (A5011, Selleck, 1:1000), CDK1 (T55176, Abmart, 1:1000), P62 (A19700, ABclonal, 1:1000), LC3B (A19665, ABclonal, 1:1000), p-ERK1/2 (AF1891, Beyotime, 1:1000), ERK1/2 (A4782, ABclonal, 1:1000), p-MEK1/2 (AP1349, ABclonal, 1:1000), MEK1/2 (A4868, ABclonal, 1:1000), GAPDH (A19056, ABclonal, 1:1000), and HRP-goat anti-rabbit IgG (AS014, ABclonal, 1:20,000).

### 4.9. RNA Sequencing

Transcriptome sequencing was performed using the Illumina NovaSeq 6000 platform (Illumina, San Diego, CA, USA). Adaptor sequences and low-quality reads were eliminated from the raw data to obtain clean reads. These clean reads were then directionally aligned to the *Homo sapiens* reference genome using the HISAT2 software (Version: 2.1.0). Differential gene expression analysis was conducted using DESeq2, applying thresholds FDR < 0.05 and FC > 2. The Majorbio platform was used to perform Gene Set Enrichment Analysis (GSEA) (https://cloud.majorbio.com, accessed on 15 April 2024).

### 4.10. ROS Detection

LX-2 cells in a six-well plate were exposed to MCL for 24 h. DCFH-DA was prepared at a concentration of 10 μM in serum-free DMEM, and 1 mL of the DCFH-DA solution was added to each well of the six-well plate, followed by a 20-min incubation. After washing the cells three times with serum-free DMEM, fluorescence intensity was measured using flow cytometry (Agilent CytoFLEX, Santa Clara, CA, USA) through the FITC channel.

### 4.11. Mitochondria Observation

After 24 h of MCL treatment, 500 μL of Mito-Tracker Red CMXRos solution (Beyotime, Shanghai, China) was added and the culture was incubated at 37 °C for 30 min. Post-incubation, the working solution was replaced with fresh culture medium. Subsequently, the red mitochondrial fluorescence was examined using a Leica confocal fluorescence microscope.

### 4.12. Mitochondrial Membrane Potential Detection

Following 24 h of MCL treatment, a JC-1 staining solution was created by combining 5 µL of JC-1 with 1 mL of JC-1 staining buffer (Beyotime, Shanghai, China). Then, 1 mL of the solution was added to each well and the plate incubated at 37 °C for 20 min. The cells were rinsed twice using JC-1 staining buffer and subsequently resuspended in 2 mL of cell culture medium before being incubated with PI stain at 10 µg/mL for 10 min, shielded from light. Mitochondrial membrane potential was detected using a flow cytometry (Agilent CytoFLEX, Santa Clara, CA, USA).

### 4.13. Molecular Docking

The TrxR1 protein structure (PDB ID: 2J3N Chains C and D) was obtained from the RCSB PDB database and prepared using Schrödinger’s Protein Preparation Wizard. Schrödinger LigPrep converted the 2D SDF of MCL into possible 3D chiral forms, and the SiteMap module identified the protein’s optimal binding site. Subsequently, the Receptor Grid Generation module established the enclosing box for the site. MCL was docked to the active site, followed by MM-GBSA calculations on the MCL-TrxR1 complex.

### 4.14. Cellular Thermal Shift Assay

LX-2 cells were treated with MCL or DMSO for 2 h and divided into four subgroups and exposed to temperatures of 55 °C, 65 °C, 70 °C, or 75 °C for 3 min. Cells were lysed by freezing and thawing three times, centrifuged at 12,000 rpm for 10 min, and then Western blotted with the supernatant.

### 4.15. Drug Affinity Responsive Target Stability

LX-2 cells cultured in a 10 cm dish were lysed using M-PER buffer to produce a protein solution which was then divided into two parts. At room temperature, both parts were incubated for one hour with MCL and DMSO, respectively. Each part was then divided into four subparts and treated with protease at ratios of 0, 1:1000, 1:2000, and 1:3000 for 2 min. The reaction was halted with 20 × protease inhibitor solution, and protein samples were analyzed using Western blotting.

### 4.16. Detection of Intracellular TrxR Activity

Intracellular TrxR activity was monitored using TRFS-green live-cell imaging. LX-2 cells were exposed to MCL for 24 h, followed by a 1-h incubation with 10 μM TRFS-green probe prior to fluorescence microscopy analysis.

### 4.17. Statistical Analysis

The images used in this study were generated by employing GraphPad Prism version 8.0.2 software (GraphPad Prism Inc., La Jolla, CA, USA). The results were presented as mean ± standard deviation (SD). Two-tailed paired Student’s t-tests were used to analyze differences between two groups. A one-way analysis of variance was used to assess statistical differences among multiple groups. A *p*-value below 0.05 was considered statistically significant.

## 5. Conclusions

In conclusion, this study showed that MCL targets TrxR1 and TrxR2 concurrently, enhancing ROS production, activating the MEK/ERK pathway, and disrupting HSC autophagic flux, which collectively inhibit HSC activation. The study demonstrates MCL’s effectiveness in inhibiting hepatic fibrosis in animal models, highlighting its potential as a candidate for preventing and treating this condition.

## Data Availability

Data is contained within the article.

## Abbreviations

MCL	Micheliolide
ROS	Reactive oxygen species
HSC	Hepatic stellate cell
ECM	Extracellular matrix
COL1A1	Collagen type I alpha 1
FN	Fibronectin
TrxR	Thioredoxin reductase
CCl_4_	Carbon tetrachloride
NAC	N-acetylcysteine
CETSA	Cellular thermal shift assay
DARTS	Drug affinity responsive target stability
GSEA	Gene Set Enrichment Analysis
HYP	Hydroxyproline
ALT	Alanine transaminase
AST	Aspartate transaminase
DMEM	Dulbecco’s Modified Eagle Medium
PBS	Phosphate buffered saline
H&E	Hematoxylin and eosin
DMSO	Dimethyl sulfoxide
GAPDH	Glyceraldehyde-3-phosphate dehydrogenase
